# Progress in Disease of Digestive Organs

**Published:** 1901-06-22

**Authors:** 


### Progress in Disease of Digestive Organs.

(*Continued from page* 183.)

**Gastric Ulcer.**—Gastric ulcer when leading to subdiaphragmatic abscess may show no symptoms of any stomach affection, and if pleurisy is produced clear fluid may be drawn off though close to the abscess. Norman Dalton[16] recently described such a case where the lung was perforated, and sputa at first smelling of lactic acid and then very fœtid, were ejected. There was a tympanitic area over the abscess which was mistaken for a displaced stomach. L. Bidwell in this discussion referred to instances where supposed empyæmata had been shown to be abscesses of this kind by the passage of food or of methylene blue from the stomach into the dressings. The detection of an opening in the diaphragm in the absence of any history of gastric ulcer may be most difficult. R. M. Matheson,[17] referring to the concurrence of gastric ulcer and appendicitis, mentions a patient who had previously suffered from ulcer and who was attacked by appendicitis. During the course of this attack hæmatemesis and symptoms of perforation preceded a fatal end. He believes that the auto-intoxication from the appendix led to acute necrosis of the stomach wall. Lauder Brunton,[18] for the pain of gastric ulcer, recommends a solution of sodium bicarbonate with lime-water and light magnesia. A large teaspoonful of soda with peppermint thus taken acts better than morphia. Sidney Martin[19] reports a case of chronic disease of the appendix, the main symptom of which was intestinal decomposition and general intoxication. Portions of food were retained in the bowels for two months in spite of diarrhœa, and at the laparotomy no obstruction of the colon could be found but a very fœtid and diseased appendix. The symptoms have, however, by other authorities been referred to ulcerative colitis which affected also the appendix.

**Cancer.**—Ehret[20] emphasises the importance for diagnosis of the discovery of Boas' bacillus filiformis, which is very long and slender, and occurs in great abundance in the fasting stomach of cancer when the motor power of the stomach is still fair. Osler in his recent work notices the presence of HCl. in a certain proportion of his cases, and perforation in four per cent. as well as occasional metastatic growths at the umbilicus and elsewhere. Early diagnosis is most important if operation is to be successful, and no palpable tumour should be present if good results are to be expected. The value of lavage is very great even if ulceration is present, and carbohydrates in the diet should be reduced to a minimum. In many cases an acute onset of the disease was noted, some patients had anorexia, but many retained a good appetite, though afraid to eat on account of the pain. The presence of blood or of scraps of malignant growth in the stomach washings is a valuable sign, but the examination of the blood cells does not aid much in diagnosis, though a count of less than a million red cells is in favour of pernicious anæmia. As in the latter disease, temporary improvement and gain of weight is sometimes met with. Boardman Reed[21] advises that treatment should be aimed at relieving the want of motor power of the stomach by light or predigested food and scanty liquids. The accompanying catarrh is aided by lavage, and the use of Condurango. Injections of a culture of Nectria have given palliative results, and seem worth further trials. Fleiner recommends the use of Chloralbacid to relieve the dyspepsia due to the absence of hydrochloric acid. W. Pasteur and Price Jones[22] report a condition of capillary stasis in the nose and feet like that in Raynaud's disease, together with a blood count showing red cells 1,460,000, and white cells 24,000 in a case of gastric cancer. The toes became black, insensitive, and showed bullæ.

**Gastroptosis.**—T. Arneill[23] finds that several viscera are often associated together in falling, and with this is present in many cases melancholia, hysterical symptoms, and chlorosis. Undue laxity of the tenth rib, dyspepsia, and palpitation are also common. Treatment comprises massage and mechanical support of the abdominal walls, the use of HCl. if subacidity is present, and nux vomica. Einhorn[24] thinks the prognosis is good, and recommends a well-fitting abdominal bandage, with supports passing round the thighs. Even though the various organs displaced do not return to their original situation, the subjective symptoms can be relieved. Digestive diseases complicating enteroptosis do not yield easily to their usual treatment. A. Rose prefers adhesive strapping, and finds that irritation of the skin can be overcome by the previous application of Unna's zinc plaster, or by using a glue formed of zinc, gelatine, and glycerine. Einhorn is opposed to operation even for the relief of the floating kidney which so often complicates enteroptosis. G. Roe Lockwood[25] lays down that gastroptosis is a predisposing cause of various neuroses, especially when mental shock occurs. Muscular atony is specially apt to occur with hyperacidity unless the latter is counteracted by gastritis. Dilatation may come on from subsequent obstruction or spasm, and for it alone surgical interference is useful.

**Carcinoma of the Duodenum.**—Rolleston[26] in describing a case mentions that only four instances were found out of 18,000 necropsies at Guy's Hospital and that it generally tends to produce an annular stricture. His patient, a plumber, showed frequent attacks of vomiting containing bile which pointed to the obstruction being below the pylorus. Much indican and creatinin was present in the urine, but no albumose. T. Bryant reports the curious phenomenon of severe melœna accompanying obstruction due to volvulus.[27] A pint of bright-coloured blood was passed in one stool and more was passed at intervals later on. A volvulus of the lower part of the ileum was found. Apparent tumours of the abdomen in the epigastric or hypochondriac regions are distinguished by M. Einhorn[28] from the phantom tumours of hysterical subjects. They are associated with enteroptosis, and may be produced by a prolapsed left lobe of the liver, an exposed and pulsating aorta, by adhesions round the lesser curvature of the stomach, or by a hypertrophic state of some of the abdominal muscles, besides cases of prolapse of entire organs. The removal of some of the pelvic viscera by operation, or great emaciation may also lead to such tumours. They give a dull note on percussion and must be diagnosed from malignant and other growths. Einhorn has seen 42 tumours of this kind in three years.

**Ascites.**—In a discussion at the Clinical Society,[29] L. Bidwell described some successful cases of treatment by laparotomy; 29 cases are recorded, of which 10 were cured. He thought that a small contracted liver counter-indicated operation. A. Morison showed that a cure could be effected in some cases by strapping on a firmly fixed many-tailed bandage. This pressure might be applied after tapping. Recently he had obtained promising results also in tuberculous peritonitis, and in cardiac ascites. Burney Yeo[30] refers to the change that has taken place in the teaching as to tuberculous peritonitis. It is now known that 50 per cent. recover, that the disease is often primary, and even the intestines may escape when the bacillus passes from the infected food through the intestinal wall to the peritoneum. He employs the inunction of iodoform whilst he gives creasote or iodoform internally, and agrees with the dictum that pleural and peritoneal tubercle is eminently curable, though in chronic or adhesive cases, and in elderly persons, the prognosis is more unfavourable. Surgical treatment is also most successful where fluid is present, but must not be done too early, or a relapse may occur. Watson Cheyne advises medical treatment for four or six weeks in acute cases, and four or six months in chronic ones before the operation is carried out. He merely opens the abdomen and allows the fluid to run out, unless it is purulent, when he washes out with saline solution, and introduces a little iodoform emulsion. The most successful cases are those where localised ascites is present, next those where it is diffused, next the dry fibrous forms, while the cases which show large caseating masses are unsatisfactory, and those where there is ulceration of the intestine even more so. Early phthisis does not counter-indicate operation.

**Appendicitis.**—H. T. Miller[31] thinks that even in America a reaction has set in against too indiscriminate surgery. He holds that if the symptoms of an acute attack subside within 48 hours we can afford to wait but if not, and if we can detect an inflammatory exudate, we must operate. He lays stress on the presence of dulness which he takes to indicate the existence of pus, and quotes a series of cases in support of this view. L. Z. Ladiniski[32] records a case of internal hæmorrhage due to rupture of adhesions by a fall during acute appendicitis. The boy suffered from cramping pains for some hours, and then fell from a staircase. Acute peritonitis came on, and when operated on the meso-appendix was found torn and bleeding freely. As a complication of appendicitis this is probably unique. Abbe[33] shows the course of the disease by distending with alcohol the appendices which he has removed and then hardening them. From these specimens it appears that strictures are first formed by contraction of small ulcers or of inflamed tissue, and that these may remain for many years before an acute attack from transient congestion takes place. Concretions form slowly beyond the stricture, which only allows fluids to pass through it, and when the stenosis is complete rupture takes place, though fatal results are often prevented by a happy perforation of the bowel, or by the formation of a localised abscess. This process can therefore only be arrested by operation, unless we accept the risk of a later attack and of results which it is impossible to foresee.

[16] Lancet, May 18. [17] Brit. Med. Jour., May 18. [18] Clin. Jour., April 24 [19] Lancet, March 18. [20] Brit. Med. Jour., March 16. [21] Merck's Archives, December. [22] Lancet, May 18. [23] Lancet, April 27. [24] Med. Rec., Jannary 5. [25] Med. Rec., December 1. [26] Lancet, April 20. [27] Loc. cit. [28] Practitioner, February. [29] Lancet, May 18. [30] Lancet, March 16. [31] Med. Rec., February 9. [32] Med. Rec., December 15. [33] Med. Rec February 16.

